# Sensitivity and specificity of HAT Bioline Abbott^®^ 2.0 and HAT Sero K-Set CORIS^®^ 2.0 for human African trypanosomiasis in the Kasai-Oriental province, Democratic Republic of the Congo: a retrospective diagnostic accuracy study

**DOI:** 10.1186/s13071-026-07504-z

**Published:** 2026-06-17

**Authors:** Simon Kayembe, Fabien Vulu, Alain Mpanya, Eddy Kakiese, Rhyno Kalonji, Olivier Fataki, Nono-Raymond Swar Kuispond, Dieudonne Mumba Ngoyi

**Affiliations:** 1 Department of Tropical Medicine, School of Medicine, University of Kinshasa, Kinshasa, Democratic Republic of the Congo; 2 National Program for the Control of Human African Trypanosomiasis, Kinshasa, Democratic Republic of the Congo; 3 Faculty of Medicine, Protestant University of Congo, Kinshasa, Democratic Republic of the Congo; 4 Department of Parasitology, National Institute of Biomedical Research, Kinshasa, Democratic Republic of the Congo; 5 Pathology Department, School of Medicine, University of Kinshasa, Kinshasa, Democratic Republic of the Congo

**Keywords:** Human African trypanosomiasis, Rapid diagnostic tests, Sensitivity, Specificity, Trypanolysis test, HAT Sero K-Set CORIS^®^ 2.0, HAT Bioline Abbott^®^ 2.0, HAT *T. b. gambiense*

## Abstract

**Background:**

Human African trypanosomiasis (HAT) due to *Trypanosoma brucei gambiense* has declined substantially in recent years, with many endemic areas now reporting very low prevalence levels as countries move toward elimination. In the Democratic Republic of the Congo (DRC), rapid diagnostic tests (RDTs) are increasingly used in surveillance and elimination programs; however, their performance in low-prevalence settings remains critical. This study evaluated and compared the diagnostic accuracy of two second-generation RDTs; HAT Bioline Abbott^®^ 2.0 and HAT Sero K-Set CORIS^®^ 2.0; using trypanolysis as the reference standard.

**Methods:**

A retrospective diagnostic accuracy study was conducted on 447 archived plasma samples (227 confirmed HAT cases and 220 controls) from a previous study in the DRC. Each sample was tested with both RDTs and trypanolysis. Sensitivity, specificity, predictive values, likelihood ratios, Cohen’s kappa coefficient, and Youden’s index were calculated with 95% confidence intervals. Paired comparisons were performed using McNemar’s χ^2^ test, with statistical significance set at *p* < 0.05.

**Results:**

HAT Bioline Abbott^®^ 2.0 showed a sensitivity of 85.2% (95% CI 80.4–90.0) and specificity of 72.2% (95% CI 66.4–77.9). The positive predictive value (PPV) was 73.1% (95% CI 67.5–78.6) and the negative predictive value (NPV) was 84.7% (95% CI 79.7–89.6).

HAT Sero K-Set CORIS^®^ 2.0 demonstrated a sensitivity of 90.0% (95% CI 85.2–93.4) and a higher specificity of 88.2% (95% CI 83.5–91.7). The PPV was 87.1% (95% CI 82.0–90.9) and the NPV was 90.9% (95% CI 86.4–93.9). HAT Sero K-Set CORIS^®^ 2.0 showed stronger agreement with trypanolysis (κ = 0.78) and a higher Youden’s index (0.78) than Abbott^®^ 2.0 (κ = 0.57; Y = 0.57).

**Conclusions:**

In this retrospective study, HAT Sero K-Set CORIS^®^ 2.0 demonstrated higher diagnostic accuracy under controlled laboratory conditions than HAT Bioline Abbott^®^ 2.0. Although both RDTs demonstrated acceptable sensitivity, neither achieved the specificity threshold (> 95%) recommended for surveillance and elimination verification in low-prevalence settings. These findings highlight the importance of optimizing specificity in serological screening tools as countries progress toward HAT elimination.

**Graphical Abstract:**

**Figure:**
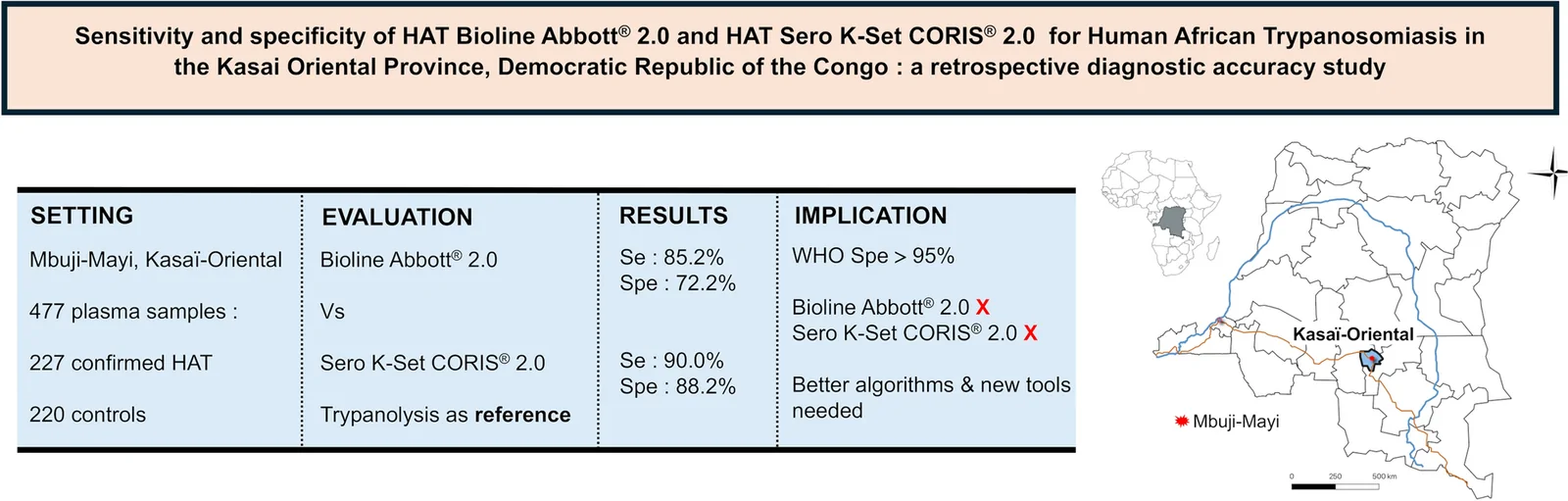

**Supplementary Information:**

The online version contains supplementary material available at 10.1186/s13071-026-07504-z.

## Background

Human African trypanosomiasis (HAT) is a fatal vector-borne disease caused by *Trypanosoma brucei* and transmitted through the bite of infected *Glossina* flies [1, 2]. The disease is confined to sub-Saharan Africa and is predominantly caused by *T. brucei gambiense* (T.b.g), responsible for the chronic form in Central and West Africa, including the Democratic Republic of the Congo (DRC), while *T. brucei rhodesiense* (T.b.r) causes the acute form in East and southern Africa [3, 4]. More than 98% of reported cases are attributable to T.b.g [1, 5, 6].

Over the past two decades, substantial progress has been made toward elimination as a public health problem (EPHP) [7, 8] Annual gambiense Human African Trypanosomiasis (gHAT) cases have declined to fewer than 2000 worldwide; however, elimination targets have not yet been fully achieved [1, 8]. By the end of 2022, only five countries had received EPHP validation, and 19 of the 24 reporting countries had not yet met the target of fewer than one case per 10,000 inhabitants per year in each health district [1, 8]. Although the DRC has reduced reported cases from 5590 in 2011 to 425 in 2021, it still accounts for nearly 70% of global cases [8]. As prevalence declines, ensuring highly accurate diagnostic tools; particularly those with high specificity; becomes increasingly critical to avoid false-positive results and unnecessary confirmatory procedures in low-prevalence settings.

Diagnosis is challenging in the early stage due to non-specific symptoms and low parasite densities, which limit the sensitivity of direct parasitological methods [2, 8]. Serological assays detecting anti-trypanosome antibodies are therefore used to identify suspects for parasitological confirmation [2, 9, 10]. For decades, screening relied on the Card Agglutination Test for Trypanosomiasis (CATT), which demonstrated sensitivities of ~ 87–95% and specificities of ~ 95–99% but required electricity, cold chain, and multi-dose vials, limiting its use in peripheral facilities [6, 11–13].

Rapid diagnostic tests (RDTs) were developed as simpler, field-adapted alternatives and are increasingly used in the DRC for both active and passive screening [7, 14]. First-generation RDTs detecting variant surface glycoproteins LiTat 1.3 and LiTat 1.5 showed sensitivities of 82–94% and specificities of 97–99% [11, 13], but reliance on native antigens raised concerns regarding production and standardization [11]. Second-generation RDTs, including HAT Bioline Abbott^®^ 2.0 and HAT Sero K-Set CORIS^®^ 2.0, incorporate recombinant antigens to improve reproducibility and scalability [11]. Multi-country evaluations confirmed feasibility, although performance varied across epidemiological contexts [15, 16], highlighting the need for context-specific validation.

In the DRC, where transmission remains heterogeneous and prevalence is declining, rigorous evaluation of second-generation RDTs is essential [11, 16]. In line with the World Health Organization Target Product Profile for gambiense HAT; which recommends minimum thresholds of > 90% sensitivity and > 95% specificity to support surveillance and elimination verification; performance must be assessed carefully in low-prevalence settings [17]

In this study, we analyzed plasma samples collected during a 2005 post-treatment follow-up cohort at the Dipumba Trypanosomiasis Reference Center (CRT) in Mbuji-Mayi, Kasai Oriental Province, DRC [4]. Using trypanolysis as the reference standard, we evaluated and compared the diagnostic accuracy of HAT Bioline Abbott^®^ 2.0 and HAT Sero K-Set CORIS^®^ 2.0 in the context of ongoing elimination efforts.

## Methods

### Study design and settings

**Fig. 1:**
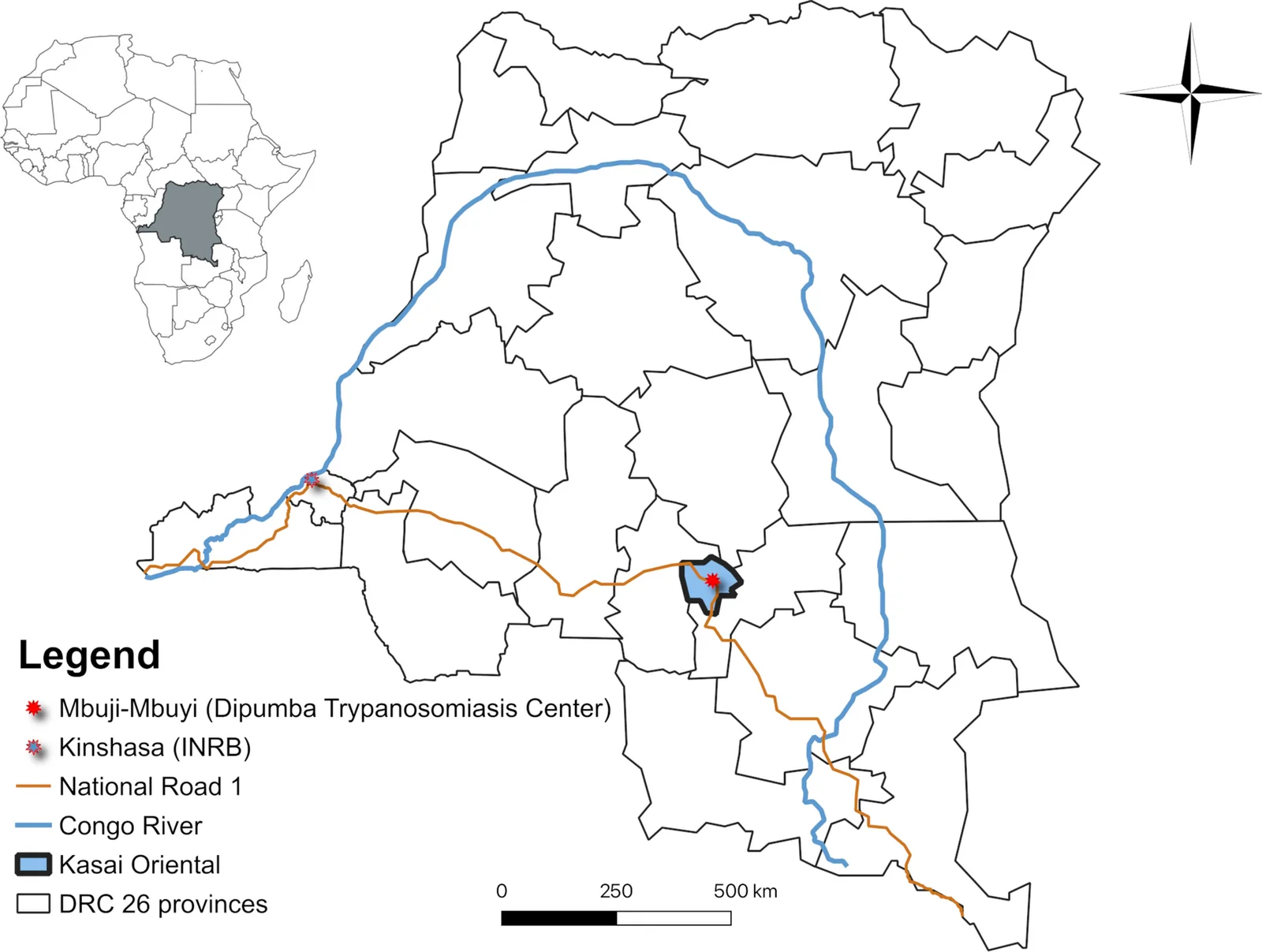
Geographic location of study settings

This was a retrospective study, reported in accordance with the STARD 2015 guidelines [18]. The study analyzed archived plasma samples originally collected in a 2005 post-treatment follow-up cohort (baseline samples at enrollment) at the Dipumba CRT, Mbuji-Mayi, Kasai Oriental Province, DRC (Fig. 1). Dipumba CRT is a specialized facility within the National Program for the Control of Human African Trypanosomiasis (PNLTHA-DRC). Samples were continuously stored at − 80 °C in a monitored cryobank at the Department of Parasitology, National Institute for Biomedical Research (INRB), Kinshasa, where all laboratory analyses were conducted between May and December 2023 (Fig. 1).

### Inclusion criteria and sample selection

- Samples were eligible for inclusion if they had been collected at baseline during the original 2005 post-treatment follow-up study, stored in cryotubes with intact and clearly identifiable labeling, and accompanied by complete clinical data and valid informed consent documentation.
- Cases were defined as individuals with parasitologically confirmed HAT by mAECT.
- Controls were defined as individuals without a history of HAT who tested negative by both CATT and mAECT at the time of collection. As trypanolysis was not performed initially, a potential risk of misclassification existed; to mitigate this, all control samples were re-tested using trypanolysis in the present study.

### Sample size

Sample size adequacy was assessed using Buderer’s formula (1996) for diagnostic accuracy studies [19]:

$$n\, = \,\frac{{Z^{2} \, \times \,Se\,\left( {1 - Se} \right)}}{{d^{2} }}$$

Assuming an expected sensitivity of 90%, consistent with the WHO Target Product Profile for gambiense HAT diagnostics, a precision of ± 5%, and a 95% confidence level (Z = 1.96), the minimum required number of confirmed cases was estimated at 139 [17, 19].

### Index tests

#### HAT Bioline Abbott^®^ 2.0

The HAT Bioline Abbott^®^ 2.0 (Abbott Diagnostics Korea Inc., Republic of Korea) is a second-generation lateral-flow immunochromatographic cassette test that incorporates recombinant antigens (rLiTat 1.5 and ISG65). The use of recombinant proteins reduces biosafety risks, simplifies production, and improves reproducibility compared with earlier tests based on native antigens [11, 15]. In this study, plasma samples were tested using cassettes from lot numbers 53FK20 and 53FK21, all within manufacturer-specified expiry dates, following the manufacturer’s instructions [11]. A volume of 10 µL of plasma was applied to the sample well, followed by 120 µL (four drops) of buffer. The cassette was incubated for 15–20 min at room temperature before reading. A valid result required the presence of a control line. The presence of one or both test lines (B1 and/or B2), regardless of intensity, indicated a positive result.

#### HAT Sero-K-SeT CORIS^®^ 2.0

The HAT Sero-K-SeT CORIS^®^ 2.0 (Coris BioConcept, Belgium) is also a lateral-flow immunochromatographic rapid diagnostic test designed to detect host antibodies against T.b.g in human blood, plasma, or serum. The test incorporates three recombinant antigens combined into a single test line: rISG65, rLiTat 1.3, and rLiTat 1.5 [13]. For this study, cassettes from lot number IFU-58S2/T1/04 (within expiry date) were used, following the manufacturer’s instructions [11, 20]. A volume of 15 µL of plasma was applied to the cassette, followed by 85 µL (two drops) of buffer. Results were read after 15–20 min. A valid test required the presence of the control line; the presence of the test line, regardless of intensity, was interpreted as positive.

#### Trypanolysis

The trypanolysis test was used as the reference standard, given its established accuracy for detecting *T.b.g*-specific antibodies [21, 22]. Briefly, 25 µL of plasma was mixed with an equal volume of guinea pig serum (as complement source), then incubated with 50 µL of trypanosome suspension (10^7^ parasites/mL) for 90 min at room temperature. Samples were examined under phase-contrast microscopy (× 250). A result was considered positive if more than 50% of trypanosomes were lysed [21].

### Test procedures and blinding

Two independent technicians, blinded to case/control status, performed the RDTs. Results were validated under the supervision of senior experts from the PNLTHA-DRC. Inter-reader variability was minimized by double reading, and discrepancies were resolved by consensus.

### Statistical analysis

Data were entered into Microsoft Excel 2019 and analyzed using SPSS version 28.0 and MedCalc version 22.032. Diagnostic accuracy metrics were calculated with 95% confidence intervals. For each RDT, we calculated sensitivity, specificity, positive predictive value (PPV), and negative predictive value (NPV), using trypanolysis as the reference standard. Comparisons of paired proportions (sensitivity and specificity between RDTs) were performed with McNemar’s chi-square test, while independent categorical variables were analyzed using the chi-square test. Agreement between each RDT and trypanolysis was assessed using Cohen’s kappa coefficient. Overall diagnostic performance was evaluated with Youden’s index, and likelihood ratios (LR) were calculated to quantify the strength of test results. A *p*-value < 0.05 was considered statistically significant.

## Results

**Fig. 2:**
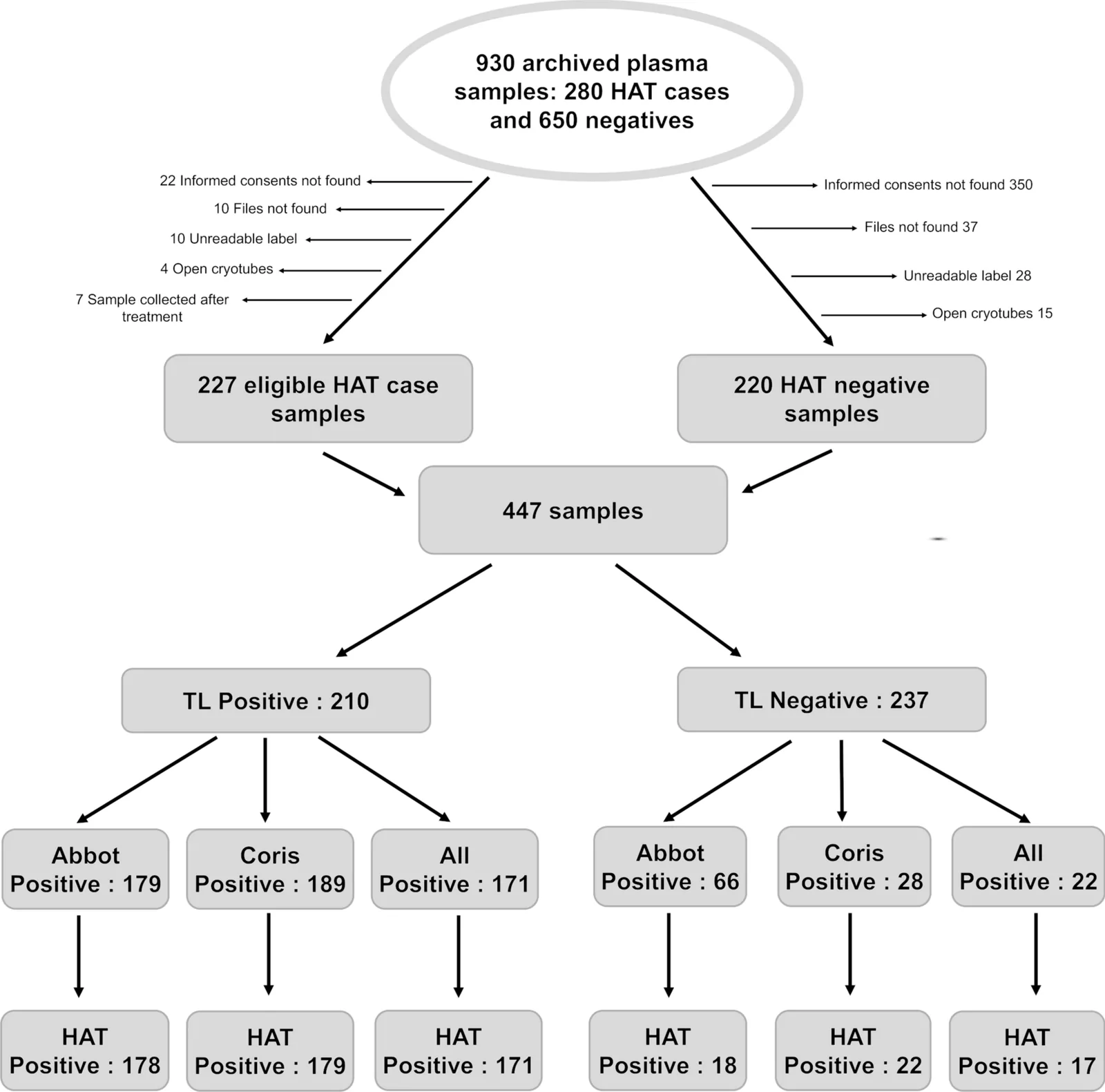
Flow diagram of sample selection and classification by reference and index tests. The diagram illustrates the selection of archived plasma samples, exclusions, and final inclusion of 447 samples. Samples were classified according to trypanolysis results (TL-positive and TL-negative), followed by the results of HAT Bioline Abbott^®^ 2.0 and HAT Sero K-Set CORIS® 2.0. “All” indicates samples that tested positive on both rapid diagnostic tests

A total of 447 samples were included in the analysis, comprising 227 confirmed cases and 220 controls (Fig. 2). The overall median age was 34 years (interquartile range [IQR]: 25–46). Cases were slightly younger than controls (median 32 vs. 38 years), and the sex distribution differed between groups, with a higher proportion of males among cases (64.7%) and females among controls (58.1%). Detailed sociodemographic characteristics are presented in Table 1.

**Table 1:** Sociodemographic characteristics of the individuals who provided the samples

Variables	Categories	Cases			Control			Total
		*n*	%	95% CI	*n*	%	95% CI	
Sex								
	Male	147	64.7	58.5 –70.9	92	41.8	35.5 – 48.4	239
	Female	80	35.3	29.1 – 41.5	128	58.1	51.6 – 64.5	208
	Total	227	100		220	100		447
Age (year)		Median: 32 (23.5 – 41)			Median: 38 (26 – 51)			Median: 34 (25 – 46)

*n* effective, *%* Percentage, *95% CI* 95% Confidence Interval

Overall, 210 of 447 samples (47.0%) were positive to trypanolysis. The corresponding positivity rates were 54.8% for HAT Bioline Abbott^®^ 2.0 and 48.6% for HAT Sero-K-Set CORIS^®^ 2.0 (Table 2).

**Table 2:** Positivity rate of the tests

Test diagnostic	*n*	positive	%	IC95%
Bioline Abbott 2.0	447	245	54.8	50.1 – 59.3
Sero- k- set 2.0	447	217	48.6	43.9 – 52.1
Trypanolysis	447	210	46.9	42.4 – 51.6

*n* effective, *%* Percentage, *95% CI* 95% Confidence Interval

HAT Bioline Abbott^®^ 2.0 demonstrated a sensitivity of 85.2% (95% CI 80.4–90.0) and a specificity of 72.2% (95% CI 66.4–77.9). The positive predictive value (PPV) was 73.1% (95% CI 67.5–78.6), and the negative predictive value (NPV) was 84.7% (95% CI 79.7–89.6). HAT Sero-K-Set CORIS^®^ 2.0 achieved a sensitivity of 90.0% (95% CI 85.2–93.4) and a significantly higher specificity of 88.2% (95% CI 83.5–91.7). The PPV was 87.1% (95% CI 82.0–90.9), and the NPV was 90.9% (95% CI 86.4–93.9). There was no statistically significant difference in sensitivity between HAT Bioline Abbott^®^ 2.0 and HAT Sero-K-Set CORIS^®^ 2.0 (McNemar’s χ^2^ = 3.1, *p* = 0.078). In contrast, specificity was significantly higher for HAT Sero-K-Set CORIS^®^ 2.0 compared with HAT Bioline Abbott^®^ 2.0 (McNemar’s χ^2^ = 27.3, *p* < 0.001) (Table 3).

**Table 3:** Diagnostic accuracy of HAT Bioline Abbott^®^ 2.0 and HAT Sero-k-set Coris ^®^2.0

Parameters	Bioline Abbott^®^ 2.0		Sero-k-set CORIS^®^ 2.0	
	Value (%)	95% CI	Value (%)	95% CI
Sensitivity (%)	85.2	80.4 – 90.0	90.0	85.2 – 93.4
Specificity (%)	72.2	66.4 – 77.9	88.2	83.5 – 91.7
PPV (%)	73.1	67.5 – 78.6	87.1	82.0 – 90.9
NPV (%)	84.7	79.7 – 89.6	90.9	86.4 – 93.9
LR +	3.1	2.5 – 3.8	7.6	5.3 – 10.8
LR-	0.21	0.15 – 0.29	0.11	0.08 – 0.17
Cohen’s Kappa coefficient	0.57	-	0.78	-
Youden’s Index	0.57	-	0.78	-

*%* Percentage, *CI* Confidence interval, *PPV* Positive predictive value, *NPV* Negative predictive value, *LR + * Positive likelihood ratio, *LR- N*egative likelihood ratio

The positive likelihood ratio (LR +) was substantially higher for HAT Sero-K-Set CORIS^®^ 2.0 (7.6; 95% CI 5.3 –10.8) than for HAT Bioline Abbott^®^ 2.0 (3.08; 95% CI 2.48–3.82). Conversely, the negative likelihood ratio (LR–) was lower for HAT Sero-K-Set CORIS^®^ 2.0 (0.11; 95% CI 0.07–0.17) compared with HAT Bioline Abbott^®^ 2.0 (0.21; 95% CI 0.15–0.29).

Agreement with the reference standard was stronger for HAT Sero-K-Set CORIS^®^ 2.0 (Cohen’s κ = 0.78) than for HAT Bioline Abbott^®^ 2.0 (κ = 0.57). Similarly, Youden’s index was higher for HAT Sero-K-Set CORIS^®^ 2.0 (0.78) compared with HAT Bioline Abbott® 2.0 (0.57).

## Discussion

This study evaluated and compared the diagnostic accuracy of two RDTs, HAT Bioline Abbott^®^ 2.0 and HAT Sero-K-Set CORIS^®^ 2.0, for the detection of T.b.g. infection in DRC, using trypanolysis as the reference standard. Both assays demonstrated similar sensitivity; however, Sero-K-Set CORIS^®^ 2.0 showed substantially higher specificity, stronger agreement with the reference standard, and improved overall diagnostic performance compared with Bioline Abbott® 2.0. Sero-K-Set CORIS^®^ 2.0 achieved a sensitivity of 90.0% and specificity of 88.2%, meeting the WHO TPP minimum sensitivity threshold but not the specificity threshold recommended for elimination verification [2, 17]. In contrast, Bioline Abbott^®^ 2.0 demonstrated slightly lower sensitivity (85.2%) and markedly lower specificity (72.2%). The higher positive likelihood ratio (LR +  = 7.6) and lower negative likelihood ratio (LR– = 0.11) observed for Sero-K-Set CORIS^®^ 2.0 indicate a stronger ability both to confirm infection when positive and to exclude infection when negative [22]. This was further reflected in its higher Cohen’s kappa coefficient (κ = 0.78) and Youden’s index (0.78), compared with κ = 0.57 and Youden’s index = 0.57 for Bioline Abbott^®^ 2.0.

Our findings align with the growing body of evidence indicating that the specificity of gHAT serological RDTs may be lower than initially reported [8, 13, 16]. Early evaluations of prototype RDTs suggested very high specificity, often above 97%, particularly in controlled diagnostic settings [2, 7]. A prospective evaluation conducted in the DRC reported high specificity for second-generation RDTs when applied within structured screening algorithms [11]. However, subsequent multi-country studies and operational evaluations have reported substantially lower specificity estimates, often ranging between 80 and 90% depending on the study design and epidemiological context [8, 13, 16]. In a large multicentric study in West and Central Africa, N’Djetchi et al. reported sensitivities of approximately 82–84% for HAT Bioline Abbott^®^ 2.0 and 78–80% for HAT Sero-K-Set CORIS^®^ 2.0, with specificities around 80% [13].

Furthermore, variable performance of serological RDTs has been reported across multiple studies and settings [8, 13, 16]. This has been described during large-scale RDT-based screening programs in Guinea, where test accuracy was influenced by operational conditions and endemicity levels [16]. Such differences likely reflect heterogeneity in epidemiological background, infection stage distribution, sample type (archived plasma versus fresh whole blood), and study design (retrospective case–control versus prospective screening) [16]. In addition, antibody persistence following infection or exposure may contribute to false-positive serological reactions and thereby reduce apparent specificity [21, 23]. These factors highlight the importance of evaluating diagnostic tools within their intended operational context and support the interpretation of our findings.

Within the context of ongoing elimination efforts, these findings are particularly relevant. The DRC continues to report the majority of global gHAT cases despite the substantial reduction in incidence observed over the past decade [2, 5, 11]. As transmission declines and prevalence falls to very low levels, the relative importance of diagnostic specificity increases, as even small decreases may generate a considerable number of false-positive results when large populations are screened. In this context, the priority shifts from maximizing sensitivity to optimizing specificity in order to reduce unnecessary confirmatory testing, associated costs, and potential misallocation of resources [23]. Compaoré et al. [23] in Burkina Faso highlighted similar challenges in monitoring HAT elimination, particularly in low-prevalence settings where sustained surveillance and accurate screening are essential. While their study focused on programmatic monitoring rather than formal diagnostic accuracy evaluation, it underscores the importance of adapting diagnostic strategies to evolving epidemiological contexts. Our findings therefore provide complementary analytical evidence on the performance of second-generation RDTs under controlled laboratory conditions.

Although both RDTs evaluated in this study demonstrated acceptable sensitivity, neither achieved the WHO-recommended specificity threshold (> 95%) required for elimination verification [2, 17]. The comparatively higher specificity observed for Sero-K-Set CORIS^®^ 2.0 suggests that it may offer advantages for surveillance programs aiming to minimize false-positive screening results and the associated burden of confirmatory testing. However, its operational performance should be confirmed through prospective evaluation in contemporary, low-prevalence field settings. These findings highlight the need for refined diagnostic algorithms that combine serological screening with highly specific confirmatory tests. Importantly, serological RDTs are intended as screening tools and cannot replace parasitological confirmation for case diagnosis [23]. In clinical and programmatic settings, confirmation of infection requires direct detection of the parasite using established parasitological techniques, together with appropriate clinical evaluation. This distinction is particularly critical in elimination contexts, where accurate case confirmation remains essential to avoid misclassification and ensure appropriate patient management. Furthermore, as the incidence of HAT declines, clinical and laboratory familiarity with the disease may decrease among healthcare providers. Maintaining adequately trained personnel, both in endemic and non-endemic settings, therefore remains essential to ensure accurate diagnosis, appropriate interpretation of test results, and sustained surveillance capacity [1, 8, 23].

The interpretation of our findings must consider the reference standard used. Trypanolysis is a highly specific serological assay for detecting antibodies against T.b.g and has been widely applied in epidemiological investigations and post-treatment follow-up studies [21, 24]. However, it reflects exposure to the parasite rather than active parasitologically confirmed infection. In elimination contexts, where individuals may remain seropositive after cure or exposure without progressing to disease, this distinction becomes particularly relevant [23]. Although trypanosome-specific antibodies have been shown to remain stable under appropriate storage conditions [21, 24], the use of a serological reference standard may influence estimates of diagnostic accuracy when compared with parasitological confirmation [25].

Furthermore, the retrospective two-gate design of this study represents an important methodological limitation. By selecting parasitologically confirmed cases and controls separately, such designs may introduce spectrum bias and do not fully reproduce the clinical and epidemiological diversity encountered during routine screening programs. As a result, diagnostic accuracy may be overestimated compared with prospective one-gate studies conducted under real-world conditions. In addition, the artificial prevalence of infection in our sample (47%) is substantially higher than that observed in elimination contexts, where prevalence is often below 1% [2, 5]. Because predictive values depend strongly on underlying prevalence, the positive and negative predictive values reported here cannot be directly extrapolated to field-based screening programs. In low-prevalence settings, even modest reductions in specificity can lead to substantial declines in positive predictive value, thereby increasing the burden of confirmatory testing and associated operational costs [26]. Consequently, while sensitivity and specificity provide robust measures of analytical performance, prospective one-gate evaluations conducted under programmatic conditions remain necessary to determine real-world diagnostic impact [23, 26].

Generalizability of the present findings is further limited by the origin of samples from a single endemic focus in the DRC and their collection in 2005. Although antibody stability supports the validity of retrospective serological analyses [21, 24] transmission dynamics, population structure, and control strategies have evolved considerably over the past two decades [5]. Therefore, the present results should be interpreted primarily as evidence of analytical performance rather than definitive guidance for current elimination policy across all endemic settings.

Despite these limitations, this study has several strengths. It included a substantial sample size exceeding the minimum required for precision [19], applied standardized laboratory procedures, and ensured blinded interpretation of index tests. In addition, the analysis incorporated likelihood ratios and agreement statistics to provide a comprehensive assessment of diagnostic performance beyond traditional sensitivity and specificity estimates.

## Conclusions

This study evaluated two second-generation RDTs, HAT Bioline Abbott^®^ 2.0 and HAT Sero-K-Set CORIS^®^ 2.0, for the detection of T.b.g infection in the DRC. Both tests demonstrated acceptable sensitivity, with HAT Sero-K-Set CORIS^®^ 2.0 showing higher overall diagnostic performance under controlled laboratory conditions but neither test reached the required specificity for elimination verification. This study provides additional evidence relevant to the use of serological screening strategies for gHAT. The findings suggest the need for optimized diagnostic strategies to meet elimination targets. Further prospective, one-gate evaluations conducted in contemporary, low-prevalence field settings are warranted to determine the real-world operational utility of these RDTs within elimination programs in DRC.

## Supplementary Information

Supplementary Material 1.

## Data Availability

All data generated during this study are included in this published article and its additional files.

## Abbreviations

CATT: Card Agglutination Test for Trypanosomiasis
CI: Confidence interval
CRT: Centre de Référence de la Trypanosomiase (Trypanosomiasis Reference Center)
DRC: Democratic Republic of the Congo
EPHP: Elimination as a Public Health Problem
gHAT: Gambiense Human African Trypanosomiasis
HAT: Human African Trypanosomiasis
INRB: National Institute for Biomedical Research
IQR: Interquartile range
LiTat: Lille Trypanosome antigen type
LR +: Positive likelihood ratio
LR –: Negative likelihood ratio
mAECT: Mini Anion Exchange Centrifugation Technique
NPV: Negative predictive value
PNLTHA-DRC: National Program for the Control of Human African Trypanosomiasis-Democratic Republic of the Congo
PPV: Positive predictive value
RDT: Rapid diagnostic test
STARD: Standards for Reporting of Diagnostic Accuracy Studies
T.b.g: *Trypanosoma* *brucei* *gambiense*
T.b.r: *Trypanosoma* *brucei* *rhodesiense*
TL: Trypanolysis
TPP: Target product profile
WHO: World Health Organization
